# Macroglossia associated with 271 bp deletion in exon 50 of dystrophin gene

**DOI:** 10.4103/0972-2327.78051

**Published:** 2011

**Authors:** Hardeep Singh Malhotra, Ratish Juyal, Kiran Preet Malhotra, Rakesh Shukla

**Affiliations:** Department of Neurology, Institute of Human Behaviour and Allied Sciences, Dilshad Garden, Delhi, India; 1 Chattrapati Shahuji Maharaj Medical University (Formerly King George’s Medical University), Lucknow, India; 2 Department of Pathology, Sanjay Gandhi Post Graduate Institute of Medical Sciences, Rae Bareilly Road, Lucknow, India

**Keywords:** Dystrophin, genetics, muscular dystrophy

## Abstract

Macroglossia is rare in patients of Duchenne muscular dystrophy (DMD), and its occurrence without any endocrinologic abnormality, seizures or an abnormal karyotype is even rarer. We describe a patient of DMD with isolated macroglossia with 271 bp deletion in exon 50 of the dystrophin gene and speculate a relationship in this regard.

## Introduction

Duchenne muscular dystrophy (DMD) is caused by frame-shift deletions, duplications or point mutations within the 79 exons of the dystrophin gene (DMD) on the X-chromosome.[1] There are several tissue-specific isoforms of dystrophin, whereby other organs besides the muscles, such as the central nervous system or the retina, may also be involved.[2] These observations, however, have not shown a one-to-one relationship and have a poor gene–protein-phenotype correlation. We describe a patient of DMD with macroglossia without any evidence of endocrinologic abnormality, seizures or an abnormal karyotype. This report highlights the possible association of macroglossia with 271 bp deletion in exon 50 of the dystrophin gene.

## Case Report

Our case is a 12-year-old son of healthy, non-consanguineous, Hindu Indian parents who presented to us with complaints of tightness and enlargement of the calf muscles since the age of 5 years. Six months after these complaints, the patient developed insidious onset and gradually progressive difficulty in standing from sitting position as well as climbing stairs. He started requiring support of at least one person for his activities of daily living by the age of 9 years and became bed-bound by the age of 10½ years. From the last 2 years, he has been having difficulty in combing hair and taking hands above the level of shoulders. Minimal difficulty in buttoning–unbuttoning and breaking chapatti is present from the last 6 months. At the age of 5 years, the parents had noted that the child’s lower jaw was quite prominent and that his tongue protruded beyond the confines of teeth. Over the next few years, drooling of saliva and difficulty in moving the food morsel inside the mouth were noticed.

There was no history of breathlessness, respiratory distress or visual or bulbar difficulties. He had been an average student before becoming bed-bound. His mother’s age was 27 years at the time of conception; pregnancy and delivery being uneventful. He was the youngest sibling, with two elder sisters; neither these nor any other relative had any similar complaints or illness.

**Figure 1:**
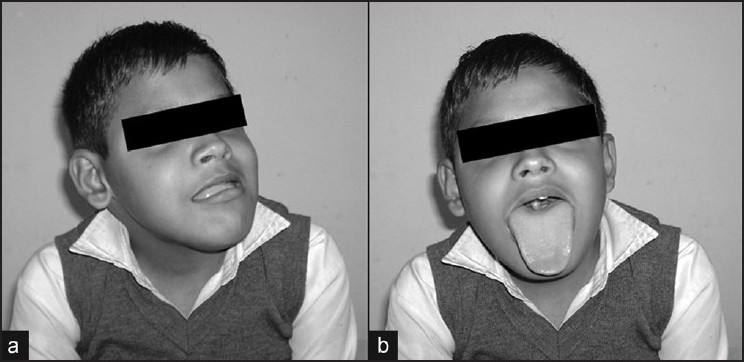
(a) Side view of the face demonstrating protruded lower jaw and improper closure of the mouth. (b) Front view of the patient demonstrating macroglossia

On examination, the child was of average stature and had a normal intelligence quotient. Lingual apex was measured to be 1.4 cm outside dentition. Impression of teeth on lateral lingual borders as well as incomplete jaw closure [Figure 1], fulfilled the criteria of macroglossia.[3] On dental examination, the maxillary and mandibular arch breadths were found to be larger than usual. Motor system examination revealed selective wasting of biceps, triceps, supraspinatus, lattisimus dorsi, pectoralis major, quadriceps and peroneii. The gastrocnemius and infraspinatus muscles were relatively preserved. Range of motion was reduced at the ankles, knees and left elbow joint, secondary to contractures. The tone was reduced at all joints. Power by Medical Research Council grade was 1/5 at the shoulder, 2/5 at the elbow, 4/5 at the wrist, 1/5 at the hip, 2/5 in the knee extensors and 3/5 in the knee flexors. Mild weakness of hand grip was present. Estimation of power at the ankles was not possible owing to the development of contractures. Moderate truncal weakness was present. Deep tendon jerks were absent with a flexor plantar response. Other neurological examination did not reveal any abnormality.

**Figure 2:**
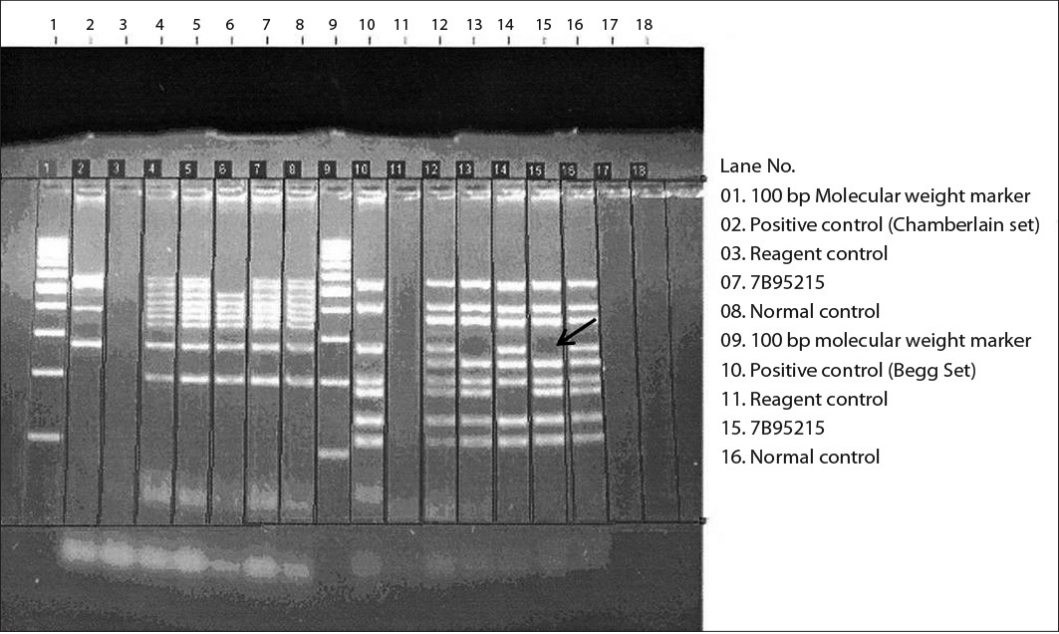
Agarose gel electrophoresis showing (arrow) absence of band in lane no. 15 when compared with normal control (lane no. 16) suggesting deletion involving exon 50

The patient had a normal hemogram and routine biochemistry. Thyroid function tests and tests for integrity of hypothalamopituitary axis were found to be within normal limits. Serum creatine phosphokinase was raised (1,670 IU). Electrophysiological studies revealed normal nerve conduction velocities with borderline low amplitudes and a myopathic pattern on needle electromyography. Ultrasound study of the abdomen did not show any abnormality. The patient was subjected to muscle biopsy and genetic testing for deletion studies along with karyotyping. Muscle biopsy of quadriceps showed muscle fiber size variation with centralization of nuclei and small basophilic groups of fibers with interspersed fibrotic areas. There was no evidence of any abnormal accumulations. Immunohistochemistry revealed complete absence of staining for dystrophin. The karyotype of the patient was found to be 46 XY with GTG banding carried out on metaphase spreads obtained from 72-h blood cultures in the RPMI 1640 medium. DNA was isolated using standard protocols. Multiplex polymerase chain reaction was used to detect deletions in the various exons of the dystrophin gene. The amplimers were subjected to agarose gel electrophoresis and visualized by ethidium bromide staining. A 271 bp deletion was observed in exon 50 with this method [Figure 2]. No additional abnormality was seen on multiplex ligation-dependent probe amplification.

## Discussion

There are several disorders affecting the skeletal muscles along with macroglossia and glycogenoses (type II), hypothyroidism, growth hormone excess, amyloidosis and muscular dystrophies constitute the important differential diagnoses. Muscular dystrophies are known for their selective involvement of muscles; DMD, Becker’s muscular dystrophy (BMD, and limb girdle muscular dystrophy type 2C[4] and 2I[5] have been shown to be associated with macroglossia. Down’s syndrome, being relatively common, must always be ruled out.

Macroglossia is a common finding in dystrophin-deficient cats,[6] but not in humans. Macroglossia and severe mental retardation have been described in a single DMD patient with a mutation at the splice donor site of the DMD intron 69.[7] It has been reported in a patient with Klinefelter’s syndrome and BMD with a homozygous deletion of *DMD* exons 45–47.[8] A recent study on videofluorographic assessment in patients with DMD has reported macroglossia as the cause of swallowing dysfunction in some patients but has not detailed the genetic abnormalities in them.[9] A patient with DMD has been described who additionally suffered from intractable seizures, severe mental retardation and a marked macroglossia. He also had endocrinologic abnormalities consisting of growth hormone deficiency, delayed puberty and adrenal hypoplasia. This patient was detected to have a duplication of *DMD* exon 18 and flanking introns that caused a frame-shift and was not removed by corrective splicing.[10]

Considering the age of the patient, phenotype and the absence of accumulations on muscle biopsy, glycogenosis type II and amyloidosis were excluded in our patient. No endocrinological abnormality was evident on evaluation. Absent dystrophin immunoreactivity in the patient’s muscle tissue ruled out limb-girdle muscular dystrophy type 2C and 2I. A normal karyotype annulled the possibility of either Down’s or Klinefelter syndrome. We wish to highlight the isolated presence of macroglossia in DMD without any other neurological or allied disorder and that 271 bp deletion in exon 50 might be contributory for this phenotypic expression. Whether this deletion defines the genetic locus abnormality leading to macroglossia or is merely contributory for this phenotypic association is conjectural. In view of the “one gene, several proteins, multiple phenotypes” hypothesis, the latter seems more plausible. A systematic evaluation is required to elucidate such associations.
